# Enhancing Chemosensitivity With Quercetin: Mechanistic Insights Into MerTK and Associated Signaling Pathways

**DOI:** 10.1002/cnr2.70370

**Published:** 2025-11-06

**Authors:** Sorush Jafari, Sorayya Ghasemi

**Affiliations:** ^1^ Cancer Research Center, Shahrekord University of Medical Sciences Shahrekord Iran; ^2^ Medical Plants Research Center, Basic Health Sciences Institute, Shahrekord University of Medical Sciences Shahrekord Iran

**Keywords:** combination therapy, efferocytosis, flavonoid, MerTK, quercetin

## Abstract

**Background:**

Quercetin, a natural flavonoid, has established significant anticancer properties through its antioxidant, anti‐inflammatory, and signaling pathway modulation effects. However, due to the issues with absorption and insufficient bioavailability, its clinical usefulness is still restricted. The purpose of this review is to review quercetin's potential as a new supplemental treatment for cancer, with a focus on the myeloid‐epithelial‐reproductive tyrosine kinase (MerTK) pathway and the downstream signaling cascades. We study ways to improve its clinical usefulness by resolving its limitations.

**Recent Findings:**

To examine research on quercetin's mechanisms of action, its impact on MerTK and downstream pathways, and the application of efficient drug delivery technologies, a thorough literature analysis was carried out. Quercetin effectively modulates MerTK‐mediated signaling pathways, reducing tumor progression, angiogenesis, and immune evasion. It suppresses PD‐L1 expression, inhibits cancer stem cell maintenance, and enhances apoptosis. Emerging evidence suggests nanoparticle‐based delivery systems can improve querecetin's bioavailability, enabling its integration into combination therapies alongside MerTK inhibitors.

**Conclusion:**

Quercetin holds great promise as a complementary therapeutic agent targeting MerTK and associated signaling pathways. Advanced delivery systems and combination strategies with MerTK inhibitors can overcome its clinical limitations and enhance its efficacy in cancer therapies. Future studies, particularly clinical trials, are needed to validate these findings and optimize quercetin's translational potential.

## 1 Introduction

Flavonoids exhibit remarkable cancer‐inhibitory effects by influencing signaling pathways, providing a wide range of beneficial properties, including antioxidant, anti‐inflammatory, and anticancer properties [1]. These compounds, divided into subfamilies, show potent chemopreventive and anti‐carcinogenic properties attributed to their rich phenolic content [2, 3]. Quercetin (3′, 3′4′, 5, 7‐pentahydroxyflavone) is a member of the flavonol group, belonging to the subclass of flavonoids. Extensive research has investigated its effects on critical biological processes, such as apoptosis induction, immune response modulation, and suppression of epithelial‐to‐mesenchymal transition (EMT) mediators. These effects may result in decreased metastasis and inhibition of angiogenesis [4, 5].

Efferocytosis is a biological mechanism that efficiently removes apoptotic cells (ACs) without inducing inflammatory responses. It plays a crucial role in immunosuppressive reactions and prevents malignant phenotypes [6, 7]. Dendritic cells (DCs) and macrophages provide fewer antigens to T cells during efferocytosis, decreasing antitumor immunity and the ability to detect malignant cells [6]. Myeloid‐epithelial‐reproductive tyrosine kinase (MerTK) is a receptor tyrosine kinase (RTK) that is expressed in monocytes, macrophages, natural killer (NK) cells, DCs, and epithelial cells of the lung, ovary, prostate, and kidney [8]. It belongs to the Tyro3‐Axl‐MerTK (TAM) family of receptors involved in recognizing ACs during efferocytosis [9]. Phosphatidylserine, a cell membrane component, acts as an “eat‐me” signal for phagocytes (efferocytes). In normal cells, phosphatidylserine is primarily located on the cytoplasmic side of the membrane. However, the preservation of phosphatidylserine asymmetry is impaired in apoptotic cells (ACs), and it is more visible on the extracellular side of the membrane in ACs than in normal cells [10].

MerTK‐dependent efferocytosis induces the secretion of anti‐inflammatory cytokines, such as IL‐4, IL‐10, IL‐13, and transforming growth factor β (TGF‐β), in macrophages and other efferocytic cells [8]. These cytokines promote tumor progression, invasion, and metastasis [11, 12]. It has been observed that IL‐4 and IL‐13 promote the survival and proliferation of cancer cells [11, 13]. This tyrosine kinase can also activate various signaling pathways, including PI3K/AKT, JAK/STAT, and RAS, which play roles in cell proliferation, survival, invasion, angiogenesis, and metastasis in many human malignancies [8].

Quercetin has been identified as a chemosensitizer capable of enhancing the efficacy of chemotherapeutic drugs against various drug‐resistant tumors [14]. Moreover, it exhibits therapeutic potential in cancer by modulating multiple signaling pathways in cancer progression. Chemotherapy‐induced inflammation caused by NF‐κB can impede cancer treatment and lead to metastasis and treatment failure [15]. Quercetin has been shown to suppress NF‐κB‐mediated inflammation through various mechanisms [16, 17]. Additionally, MerTK inhibitors may have specific limitations. In this context, quercetin emerges as an up‐and‐coming option to overcome these drawbacks and be utilized with MerTK inhibitors.

**FIGURE 1:**
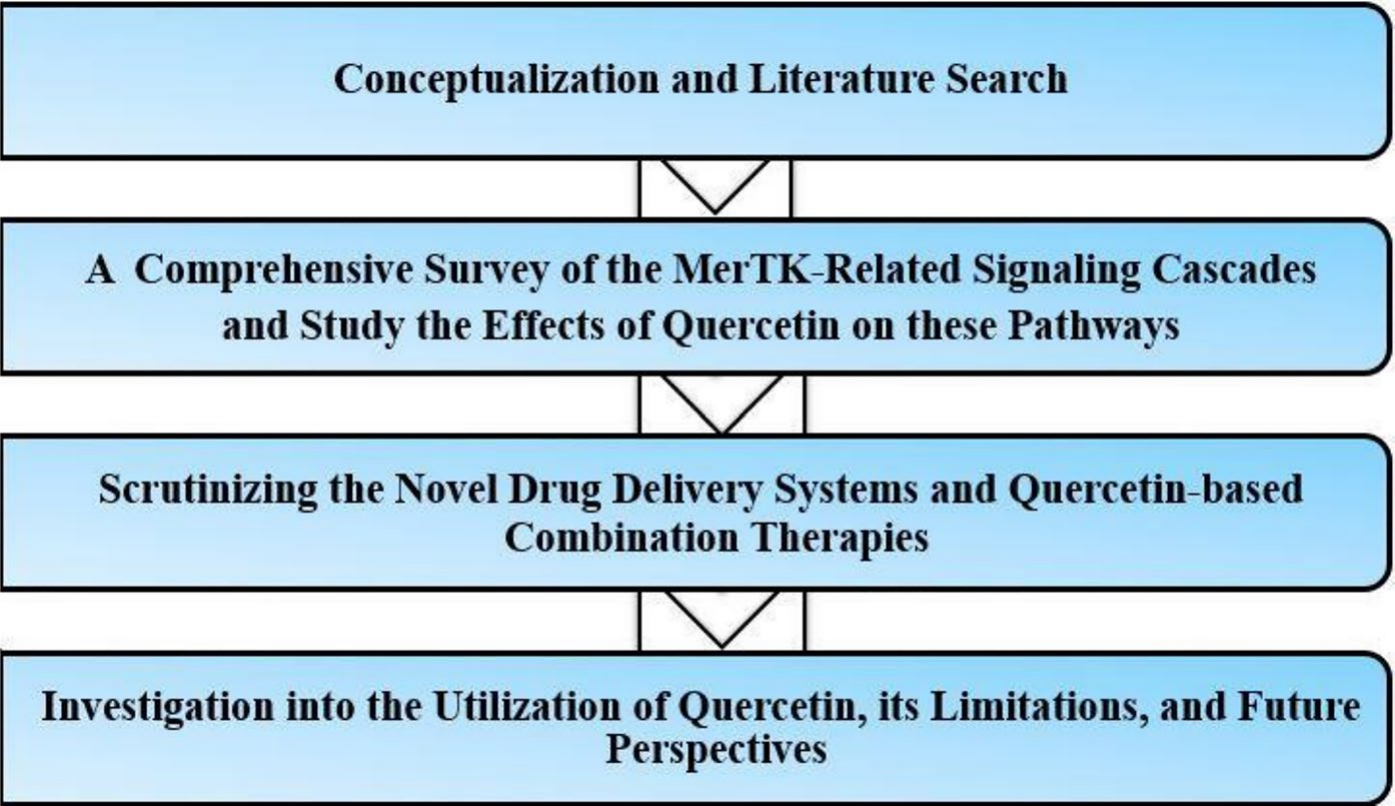
An overview of the study. This schema shows the different sequential steps of this study.

Recent developments in drug delivery technologies, such as nanoparticles, have shown tremendous promise in overcoming quercetin's therapeutic limitations. Furthermore, quercetin may be more clinically effective, less resistant to drugs, and more effective for patients when it is used through developed drug delivery systems and/or in combination with targeted treatment agents such as MerTK inhibitors. In order to demonstrate quercetin's potential in cancer treatments, this review examines related molecular mechanisms, drug delivery systems, and relevant combination therapies. An overview of this study is shown in Figure 1.

## 2 Methods of Literature Search

For the purpose of this review, a systematic search was conducted in reputable scientific databases including PubMed, Scopus, and Web of Science. The search period was considered from 2005 to 2025. The used keywords were: “Quercetin,” “MerTK,” “TAM receptors,” “Efferocytosis,” “Tyrosine kinase,” “Cancer,” “and Nanotechnology,” “TKIs,” and “Combination therapy.”

Inclusion criteria included laboratory research articles (in vitro and in vivo), clinical trials, and review articles related to the role of quercetin in cancer signaling pathways and treatment. Articles that only discussed plant compounds without a direct relationship with quercetin or the MerTK pathway, articles in non‐English languages, and articles without valid scientific data were excluded from the study.

## 3 Targeting MerTK as a Key Candidate in Cancer Therapies

Numerous recent studies have been conducted using monoclonal antibodies (mAb) and tyrosine kinase inhibitors (TKIs) to inhibit TAM receptors, such as MerTK (Table 1). Blockade of MerTK can suppress the survival, invasion, angiogenesis, and metastasis of tumor cells.

**TABLE 1:** Examples for MerTK inhibitors: introducing potential MerTK‐targeting compounds for cancer therapies.

Type	Name	Target(s) (in TAM family)	References
Monoclonal antibody	**AF591**	**MerTK**	[18]
	**Mer590**	**MerTK**	[19]
	**RGX‐019**	**MerTK**	[20, 21]
Tyrosine kinase inhibitor	**6 g**	**MerTK/Axl/Tyro3**	[22]
	**AZ14145845**	**MerTK**	[23]
	**Compound‐52**	**MerTK**	[8]
	**CT413**	**MerTK/Axl**	[24]
	**INCB081776**	**MerTK/Axl**	[25, 26]
	**MRX‐2843**	**MerTK**	[27]
	**ONO‐7475**	**MerTK/Axl**	[28]
	**PF‐07265807**	**MerTK/Axl**	[29]
	**RXDX‐106**	**MerTK/Axl/Tyro3**	[30]
	**Sitravatinib**	**MerTK/Axl/Tyro3**	[31]
	**UNC1062**	**MerTK/Axl/Tyro3**	[32]
	**UNC2025**	**MerTK/Axl/Tyro3**	[33, 34]
	**UNC2250**	**MerTK**	[35]
	**UNC2541**	**MerTK/Axl/Tyro3**	[36]
	**UNC3133**	**MerTK/Axl/Tyro3**	[37]
	**UNC5293**	**MerTK/Axl/Tyro3**	[38]
	**UNC569**	**MerTK**	[39]
	**XL092**	**MerTK/Axl**	[40]

TAM receptors consist of two Ig‐like domains and two fibronectin type III repeats in the extracellular region, a single‐pass transmembrane domain, and an intracellular (cytoplasmic) kinase domain [41]. This family of receptors exhibits 52%–57% similarity in their extracellular regions, while the intracellular regions demonstrate 72%–75% similarity, specifically within the tyrosine kinase domain [42]. Due to the structural similarity of the TAM receptor family, usage of MerTK inhibitors in anti‐cancer approaches can result in elevated off‐target effects and toxicity.

## 4 Evidence of the Intervention of Quercetin With MerTK‐Mediated Efferocytosis and TGF‐β

**FIGURE 2:**
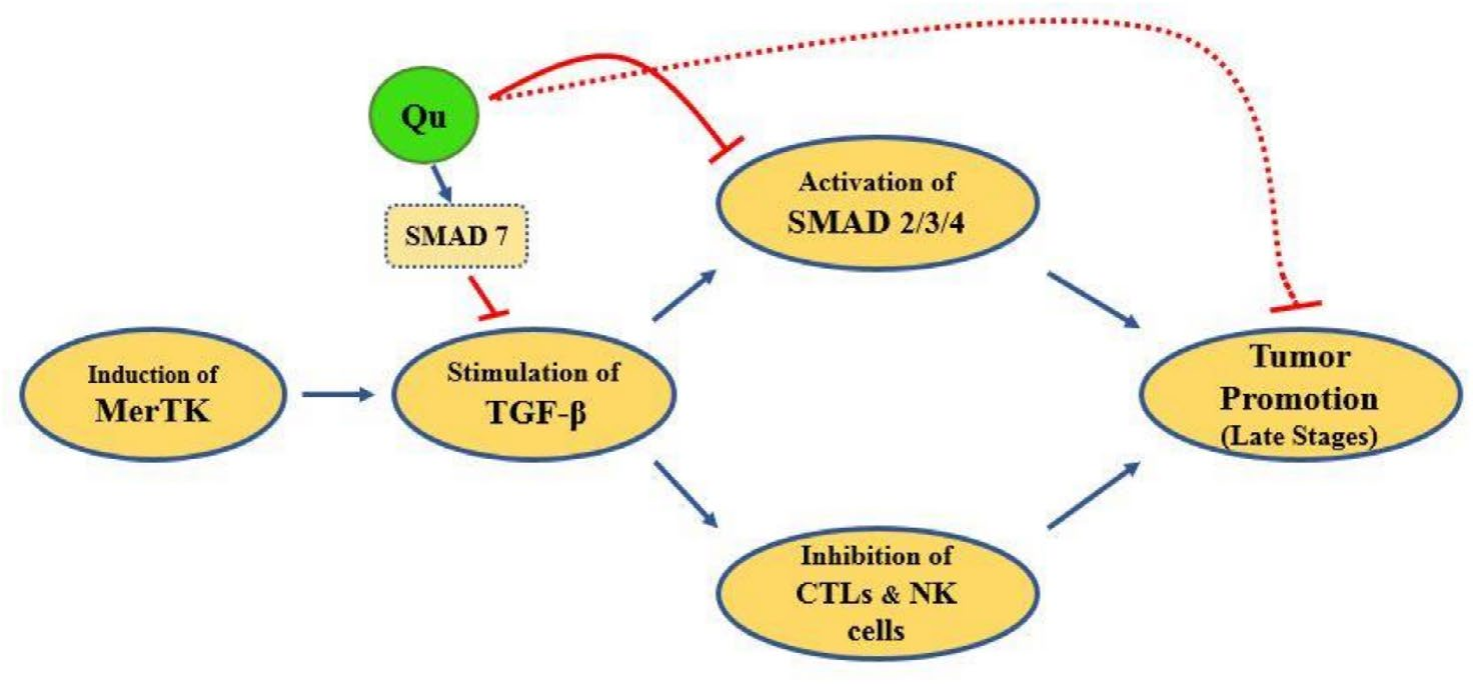
Effects of quercetin on MerTK‐related stimulation of TGF‐β. Quercetin affects the downstream events of the TGF‐β stimulation through different mechanisms including the activation of SMAD7, reduction of SMAD 2/3/4 levels, and suppression of tumor promotion via indirect procedures.

Transforming growth factor β (TGF‐β) is a multifunctional and anti‐inflammatory cytokine that is secreted through the induction of MerTK‐dependent efferocytosis [43]. TGF‐β acts as a tumor promoter factor in the late stages of malignancy. This factor also inhibits certain functions of cytotoxic T lymphocytes (CTLs) and natural killer cells [44] (Figure 2). TGF‐β induces tumor‐associated angiogenesis and is associated with cancer invasion and metastasis. In addition, its signaling pathway is vital for maintaining CSCs [45].

In a survey of mesangial cells, quercetin reduced TGF‐β1, SMAD2, SMAD3, and SMAD4 levels. Interestingly, quercetin upregulated the expression of SMAD7, which acts as a negative regulator in the TGF‐β pathway in these mesangial cells [46]. Another study focusing on pancreatic ductal adenocarcinoma reported that quercetin reduces the activity of the TGF‐β1/SMAD2/3 signaling pathway. Quercetin decreased the expression of TGF‐β1 and inhibited the phosphorylation (activation) and nuclear translocation of SMAD2/3. Moreover, quercetin reduced the expression of Snail1 and Zeb2 (as two target molecules in the TGF‐β1/SMAD2/3 signaling pathway), transcription factors that induce epithelial‐mesenchymal transition (EMT) [47]. Another study on LX‐2 cells, a hepatic stellate cells (HSCs) cell line, demonstrated that quercetin suppressed the activation of HSCs by inhibiting the TGF‐β/SMAD3 signaling pathway [48]. A study on rats indicated that quercetin downregulated TGF‐β1 and SMAD3 and suppressed the phosphorylation of SMAD3 in both control and myocardial infarction (MI)‐induced rats. Furthermore, it upregulates SMAD7 expression, which leads to the increased inhibition of the TGF‐β signaling pathway [49]. These studies collectively provide robust evidence supporting the potential of quercetin as an effective regulator of the TGF‐β signaling pathway in various cellular contexts and disease conditions.

## 5 Evidence of the Intervention of Quercetin With MerTK‐Mediated Efferocytosis and PD‐1/PD‐L1

**FIGURE 3:**
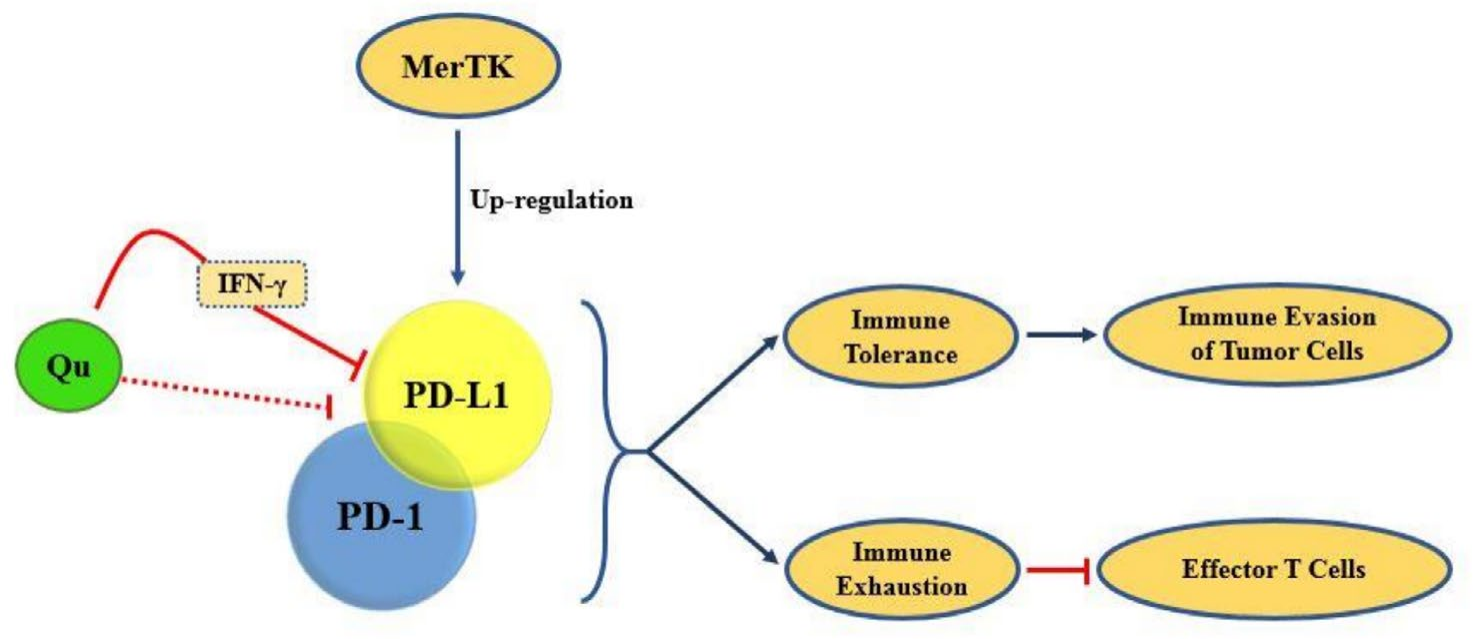
Intervention of quercetin on the PD‐1/PD‐L1 interaction and the sequential processes. Quercetin can suppress the immune evasion of tumor cells and hinder the impairment of effector T cells through interfering with the PD‐1/PD‐L1 interaction and IFN‐γ‐mediated induction of PD‐L1's expression.

The interaction between PD‐1 and PD‐L1 is critical for immune tolerance and exhaustion. Immune exhaustion refers to the functional impairment of effector T cells. In malignancies, the overexpression of PD‐L1 in malignant cells is essential for preventing the immune system from destroying them. Interaction of PD‐1/PD‐L1 upregulates MAPK/ERK‐mediated expression of Slug, Snail, and Twist, which may lead to invasion [50] (Figure 3).

It has been indicated that TAM receptors, including MerTK, upregulate the expression of PD‐L1 in a breast cancer cell line [51]. Additionally, MerTK‐mediated efferocytosis promotes osteosarcoma progression by enhancing PD‐L1‐induced immunological tolerance and M2 polarization of macrophages. Efferocytosis increases the expression of PD‐L1 in macrophages [52].

Quercetin and formononetin have shown promising results in inhibiting PD‐1 [53]. Interferon‐gamma (IFN‐γ) is a potent inducer of PD‐L1 expression. It has been shown that quercetin suppresses the IFN‐γ‐induced upregulation of PD‐L1 [54]. Released quercetin from the quercetin‐Ferrum ion (QFN) suppresses the phosphorylation of JAK2/STAT3 and reduces PD‐L1 expression in tumor cells. A reduction in PD‐L1 expression in tumor cells enhances the activity of cytotoxic T cells (CTLs) [55]. In other words, quercetin inhibits JAK2/STAT3/PD‐L1, thereby preventing the immune escape of tumor cells. A decrease in PD‐L1 levels is linked to increased quercetin concentration [56]. In addition, quercetin dihydrate inhibits the interaction of PD‐1/PD‐L1 in two cell lines of breast and lung cancer [57]. In conclusion, quercetin appears to be a promising choice for inhibiting MerTK‐mediated efferocytosis and PD‐1/PD‐L1 interaction.

## 6 MerTK‐Mediated JAK/STAT, PI3K/AKT, NF‐κB, RAS, and Src Signaling Pathways and Interventions by Quercetin

**FIGURE 4:**
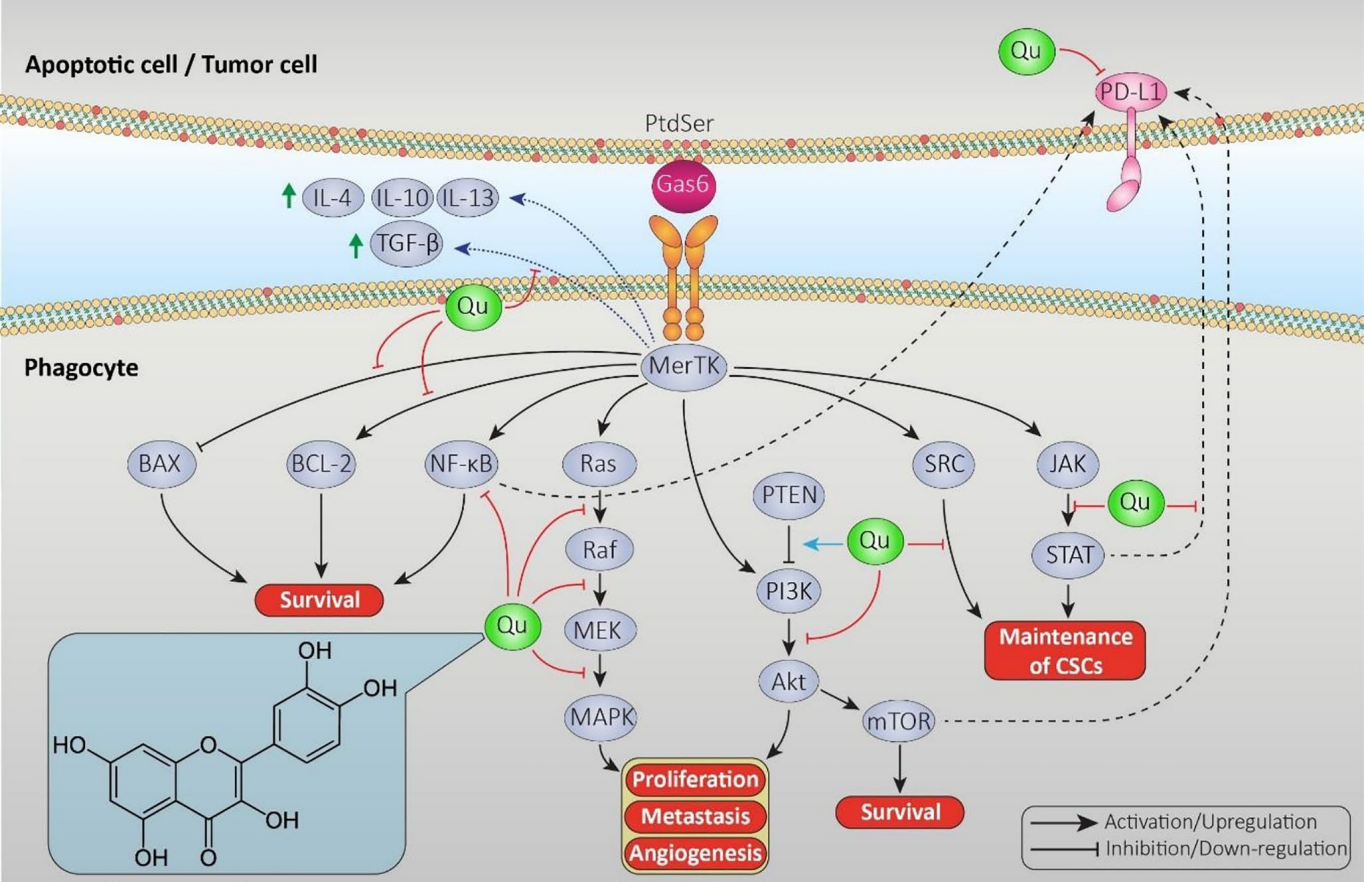
Effects of Quercetin on different MerTK‐related signaling pathways. The antitumor effects of Quercetin on several MerTK‐related signaling cascades can lead to reduced maintenance of CSCs and exhibit an inhibitory role in proliferation, metastasis, and angiogenesis of tumor cells through various mechanisms.

Efferocytosis and its downstream molecular pathways lead to the secretion of anti‐inflammatory cytokines and the recognition of ACs. Besides these, downstream pathways of MerTK‐mediated efferocytosis, such as JAK/STAT, PI3K/AKT, NF‐κB, RAS, and SRC, are essential for cell proliferation, survival, invasion, angiogenesis, and metastasis. Moreover, blocking MerTK, other TAM receptors, or their ligands may result in excessive inflammation and the evasion of tumor cells from undergoing apoptosis. Meanwhile, quercetin can cause a reduction in inflammatory mediators (e.g., IL‐2, IL‐6, TNF‐α, and COX‐2), stimulation of intrinsic pathways of cell death, inhibition of anti‐apoptotic factors, and stimulation of pro‐apoptotic factors [53]. With its recognized advantages, quercetin may be a beneficial option in cancer combination therapy by targeting MerTK. It can be used independently or in combination with MerTK inhibitors, depending on the limitations associated with MerTK inhibitors (Table 2) (Figure 4).

**TABLE 2:** Mechanism and effect of quercetin on different cell lines and cancers within MerTK‐associated signaling pathways.

Signaling pathway	Type of cancer or cell	Cell line	Mechanism	References
JAK/STAT	Melanoma	A375 A2058	↑Apoptosis ↓Angiogenesis ↓Invasion ↓Cell growth	[58]
	Cholangiocarcinoma	KKU100 KKU‐M139 KKU‐M213	↓Migration ↓Cell growth	[59]
	Squamous carcinoma	A431‐P A431‐III	↓Migration ↓Invasion	[60]
	Hepatocellular carcinoma	LM3	↓Cell proliferation ↓Invasion ↓Migration ↑Apoptosis ↑Autophagy	[61]
	Microvascular endothelial cells	HMEC‐1	↓Migration ↓Tube formation	[62]
PI3K/AKT	Gastric CSCs	MGC803	↓Cell survival ↑Apoptosis	[63]
	Primary effusion lymphoma (PEL)	bc1 bc3 BCBL1	↓Cell survival ↑Cytotoxicity ↓ IL‐10 and IL‐6	[64]
	Breast cancer	MCF‐7 MDA‐MB‐231	↑Apoptosis ↓Cell growth	[65]
	Breast CSCs	MCF‐7	↓Clone formation ↓Cell viability ↓Metastasis ↓Cell growth ↑Apoptosis	[66]
NF‐κB	Coronary artery disease (CAD)	—	↓IL‐1β, IL‐10, and TNF‐α ↓Inflammation	[67]
	Human gingival fibroblasts (HGFs)	—	↓ IL‐1β, IL‐6, IL‐8, and TNF‐α ↓Inflammation	[68]
	Lung epithelial cells	A549	↓Oxidative stress ↓Inflammation	[16]
RAS	Ovarian cancer	PA‐1	↓Cell proliferation ↓Cell survival ↓Migration ↓Metastasis	[69]
	Liver cancer	MHCC97H Hep3B HCCLM3 Bel7402	↓Cell proliferation ↓Colony formation ↓Migration ↑Apoptosis	[70]
SRC	Cardiomyocytes	H9C2	↓Oxidative stress ↓Inflammation	[71]
	Squamous carcinoma cells	A431‐P A431‐III	↓Migration ↓Invasion ↓Metastasis	[60]

### 6.1 Jak/Stat

Abnormal regulation of STAT3 results in tumor invasion, angiogenesis, and metastasis. It also helps malignant cells evade apoptosis by increasing the expression of the inhibitor of apoptosis (IAP). P53 is a tumor suppressor factor that plays a significant role in apoptosis and the regulation of proliferation. STAT3 can bind to the promoter of P53 and reduce its transcription [72]. The STAT3 pathway is a downstream target of MerTK signaling [73]. Therefore, the upregulation of MerTK results in abnormal levels of JAK/STAT. The phenotype of CSCs is maintained through the mediation of JAK/STAT3/SRC downstream of MerTK signaling [74].

Quercetin's inhibitory effects on the JAK/STAT pathway have been studied in various malignancies [75]. In one study, it was found that quercetin has anti‐melanoma activity. Quercetin inhibits the activation of STAT3 by impeding the phosphorylation of STAT3 and decreasing the nuclear localization of STAT3. Continuous activity of JAK/STAT3 stimulates angiogenesis, invasion, and metastasis. Inhibiting the transcription activity of STAT3 results in the down‐regulation of its downstream targets, including matrix metalloproteinase‐2 (MMP‐2), MMP9, and vascular endothelial growth factor (VEGF). In addition, quercetin induces apoptosis and inhibits the invasive capacity of melanoma cells. Interestingly, overexpression of STAT3 suppresses cell growth inhibition by quercetin [58]. In a study on cholangiocarcinoma (CCA), quercetin and epigallocatechin‐3‐gallate (EGCG) showed inhibitory effects on the JAK/STAT pathway of CCA cells [59]. Treatment of a human squamous carcinoma cell line with quercetin and luteolin resulted in a decrease in protein levels of p‐STAT3 and a reduction in the migratory and invasive abilities of the cells [60]. Hepatocellular carcinoma LM3 cells treated with quercetin show suppression of the JAK2/STAT3 signaling pathway. Treatment of the cells with quercetin shows a reduced expression of p‐STAT3 [61]. In a study on the treatment of human microvascular endothelial cells with quercetin, inhibition of cell migration and tube formation by suppressing the JAK2/STAT3 and PI3K/AKT pathways through modulation of miR‐216a was observed [62].

### 6.2 PI3K/Akt

Activation of the MerTK/PI3K/AKT signaling pathway by apoptotic cells caused by chemotherapy or hypoxia promotes the survival of neighboring cells due to the anti‐apoptotic properties of this pathway [76].

Quercetin inhibits the PI3K/AKT pathway and upregulates pro‐apoptotic factors, which reduces cell survival and increases tumor cell apoptosis [77]. Gastric cancer stem cells show promising results after treatment with quercetin. Quercetin suppresses cell survival and induces apoptosis in gastric CSCs. Inhibition of the PI3K/AKT pathway, activation of caspase‐3 and caspase‐9, upregulation of Bcl‐2‐associated X protein (BAX), and downregulation of BCL‐2 have been observed in gastric CSCs [63]. It has been indicated that treating primary effusion lymphoma (PEL) cells with quercetin inhibits the PI3K/AKT and STAT3 pathways. This inhibition leads to a decrease in the expression of pro‐survival cellular proteins and results in cytotoxicity in PEL cells. Additionally, the release of IL‐6 and IL‐10 decreases after treatment with quercetin [64]. Treatment of CD44+/CD24‐ breast CSCs with quercetin leads to suppression of the PI3K/AKT/mTOR pathway. In addition, clone formation, cell viability, and metastasis ability of breast CSCs are inhibited. CyclinD1 and B‐cell lymphoma‐2 (BCL‐2) are other suppressed factors, while BAX is an enhanced factor in breast CSCs after treatment with quercetin [66]. Since CyclinD1 is a positive regulator of the cell cycle, BCL‐2 is an anti‐apoptotic factor [78], and BAX is a pro‐apoptotic factor [79], quercetin may act as an inhibitor of cell proliferation and a stimulator of apoptosis in breast CSCs. On the whole, quercetin can constrain PI3K/AKT/mTOR signaling cascade in brain, breast, colon, pancreatic, and prostate cancers [80].

### 6.3 NF‐κB

In the cytoplasm, nuclear factor kappa B (IκB) inhibitors bind to NF‐κB and prevent the dimerization of this factor. Phosphorylation of IκBs induces their ubiquitination and degradation. As a result, the released dimers of NF‐κB enter the nucleus to upregulate or downregulate different genes [81]. Various cytokines, growth factors, and tyrosine kinases can activate NF‐κB. Other signaling pathways, such as PI3K/AKT and RAS/MAPK, can also activate NF‐κB. This factor also decreases the expression of PTEN. Activation of FLIP and IAPs, along with down‐regulation of PTEN, leads to the suppression of apoptosis. Thus, the upregulation of NF‐κB is responsible for apoptosis resistance in both intrinsic and extrinsic cell death pathways. VEGF, ICAMs, and matrix metalloproteinases are other factors that NF‐κB promotes. Moreover, NF‐κB regulates the expression of cyclins D1, D2, D3, and E, which can contribute to the progression of the cell cycle and cell proliferation. Constitutive activation of NF‐κB is associated with resistance to chemotherapy and radiotherapy in many tumors [82]. NF‐κB is a crucial downstream target of MerTK and can be activated by this tyrosine kinase [83, 84].

In patients with coronary artery disease (CAD), quercetin has decreased the transcriptional activity of NF‐κB and reduced serum levels of IL‐1β [67]. In a study on the anti‐UV effects of quercetin, researchers demonstrated that quercetin‐loaded nanoparticles (TTP‐Chitosan or TC) decreased the phosphorylation of IκB and enhanced the inhibitory effects of quercetin on NF‐κB [85]. Additionally, quercetin has been shown to inhibit the NF‐κB pathway in human gingival fibroblasts (HGFs), reducing the production of IL‐1β, IL‐6, IL‐8, and TNF‐α [68]. Intriguingly, quercetin significantly inhibits the degradation of IκBα and the nuclear translocation of NF‐κB in lung epithelial cells [16]. Additionally, treatment with quercetin has been shown to increase mRNA levels of IκB, leading to the downregulation of NF‐κB and the inhibition of the NF‐κB signaling pathway [17].

### 6.4 Ras

The RAS signaling pathway is a key downstream target of MerTK. Additionally, TGF‐β, which can be activated by MerTK‐dependent efferocytosis, can activate the RAS signaling pathway [41]. MerTK activates the STAT3/KRAS and SRC signaling cascades in glioblastoma multiforme (GBM). Also, it has been demonstrated that the STAT3/KRAS and SRC signaling pathways are essential for maintaining stemness in glioma cells [73].

Evidence shows that quercetin can affect the RAS signaling pathway [86]. Quercetin stimulates the degradation of the KRAS and HRAS oncoproteins, and it can also inhibit the downstream effectors of the RAS signaling pathway, such as Raf and MEK [87]. The RAS and PI3K/AKT signaling pathways are activated in ovarian cancer, leading to the increased survival, proliferation, and metastasis. A study on human metastatic ovarian cancer revealed that quercetin inhibits the RAS and PI3K/AKT pathways and suppresses the expression of EGFR. According to these findings, quercetin may inhibit the proliferation, survival, migration, and metastasis of malignant cells in metastatic ovarian cancer in humans. The expression of NRAS and Raf‐1 is decreased due to treating cells with quercetin [69]. Surprisingly, a structural study demonstrated that quercetin can directly bind to the Raf protein, a downstream target of Ras, and suppress its function [88].

### 6.5 Src Family Tyrosine Kinases

Src, a non‐receptor tyrosine kinase, is a member of the Src family of tyrosine kinases (SFKs) [89]. Src is indirectly involved in tumor formation. Thus, inhibiting Src as a monotherapy is not an ideal choice, but it should be considered a significant option in cancer combination therapies [90]. MerTK regulates cell migration, phagocytosis, and actin rearrangement through signaling pathways such as Src/FAK/Rac1. The binding of MerTK to its ligand, Gas6, leads to the activation of Src. Subsequently, Src phosphorylates FAK [9]. Additionally, MerTK activates the FAK/SRC‐STAT3 signaling pathway through phosphatidylserine‐induced macrophage polarization [91]. Also, the inhibition of MerTK by UNC2025 decreases SRC and AKT levels [92].

In a study on cardiomyocytes, it has been indicated that quercetin inhibits Src, STAT3, p38, and FAK in these cells. Therefore, besides its anticancer effect by inhibiting the Src signaling pathway, quercetin demonstrates cardioprotective effects in the H9C2 cell line [71]. Quercetin suppressed the activation of Src, PI3K, and AKT in a manner dependent on both time and dose. In other words, quercetin inhibits the lipopolysaccharide‐induced activation of Src in a concentration‐dependent manner [93]. It has been suggested that quercetin can directly bind to the ATP‐binding site of the Src family and modulate cell signaling [94]. Moreover, studies have demonstrated that quercetin inhibits the Src signaling pathway in squamous cell carcinoma. Quercetin decreases the migration and invasion of squamous carcinoma cells by reducing the p‐Src and p‐Stat3. Above all, the treatment of tumor cells with quercetin leads to the inhibition of EMT signaling and, thereby, suppresses the metastasis of tumor cells in vivo by inhibiting the Src/Stat3 signaling pathway [60].

## 7 Cancer Stem Cells, MerTK, and Quercetin

Cancer stem cells (CSCs) are considered to be the instigators of tumor initiation, resistance, metastasis, and recurrence, and they are believed to play a central role in tumorigenesis [95].

PD‐L1 is overexpressed in CSCs, which is essential for the immune evasion of these cells. PD‐L1 expression is also associated with the expression levels of CSC markers in colorectal [96] and pancreatic cancer cells [97]. Moreover, the expression levels of PD‐L1 are increased in endometrial CSCs and are induced by hypoxia. Knockdown of PD‐L1 suppresses the expression of various pluripotency‐related genes, including ALDH1, CD133, OCT4, SOX2, and NANOG. Blockade or knockdown of PD‐L1 also correlates with decreased tumorigenicity of endometrial CSCs [98]. Quercetin has been shown to exhibit inhibitory activities against the interactions between PD‐L1 and its receptor, PD‐1, as well as other signaling pathways that activate PD‐L1.

Furthermore, the maintenance and stemness of CSCs seem to depend on various signaling pathways, including JAK/STAT, PI3K/AKT, NF‐κB [99], RAS [100], and SRC [101]. Quercetin suppresses all of these signaling pathways through various known and unknown mechanisms (Figure 4). In addition to constraining the growth of CSCs through several molecular mechanisms, quercetin has the aspects of enhancing the anti‐neoplastic activities of chemotherapeutic agents including paclitaxel, doxorubicin, and vincristine [102].

Thus, abnormal activation of MerTK can promote the stemness phenotype of malignant cells, including CSCs. On the contrary, quercetin appears to have therapeutic effects against CSCs in various procedures and can be considered a suitable option for combination therapies with other anticancer agents.

## 8 Nanoparticles and Cancer Combination Therapies Using Quercetin Can Increase Quercetin Utilization Efficiency

### 8.1 Nanoparticles and Quercetin

Despite its antioxidant, anti‐inflammatory, and anticancer properties, quercetin has some drawbacks that can be managed using new methods and technologies. The main limitation of free quercetin is its inadequate aqueous solubility, which is caused by the hydrophobic nature of this substance [103]. Furthermore, bioactive compounds like quercetin have limited chemical stability and may be rapidly eliminated in their free forms [104]. The poor permeability of quercetin can be considered as another disadvantage [105]. Consumption of high doses or uncontrolled release of quercetin may cause specific issues in the body [106].

To overcome the challenges related to hydrophobic anticancer drugs such as quercetin, drug carriers, specifically nanoparticles, are employed. Numerous studies have demonstrated that nanoparticles can increase the efficacy of these compounds and overcome their limitations [107]. This is achieved by enhancing absorption and permeability, improving the maintenance and stability of bioactive compounds, and controlling the release of quercetin in the body [108]. The controlled release is considered an essential advantage of nanoparticles. Additionally, drug‐coated nanoparticles are easy to produce and relatively low‐cost. Interestingly, drug‐coated nanoparticles can be absorbed by endothelial cells of the skin, enabling improved topical delivery. They can also penetrate the pulmonary system and cross the blood–brain barrier (BBB), enabling drug delivery to target specific tissues and providing potential therapeutic benefits for diseases involving the central nervous system [104]. In a study on breast cancer cells, gold nanoparticles (AuNP) conjugated with quercetin showed promising results. Cells treated with AuNP‐Qu exhibit more significant suppression of EGFR and PI3K/AKT/mTOR levels than free quercetin. Moreover, compared with free quercetin, AuNP‐Qu demonstrates a more efficient induction of apoptosis and upregulation of PTEN (a tumor suppressor factor) [65]. Additionally, quercetin gold nanoparticles (Qu‐AuNPs) decrease Raf, p‐ERK1/2, and p‐Akt levels in liver cancer cells. Induction of apoptosis and suppression of cell proliferation, migration, and colony formation are other advantages of Qu‐AuNPs in liver cancer [70].

Moreover, quercetin has chelating properties concerning metal ions such as Fe^2+^, Fe^3+^, Cu^2+^, and Ni^2+^ [105]. One of the most significant advantages of nanoparticles is their ability to enhance and target the accumulation of drugs in tumors [4]. Nanocarriers can include dendrimers, liposomes, polymeric micelles, carbon nanomaterials, and metal oxide nanoparticles [103]. Chitosan nanoparticles (CS‐NPs) are commonly used as carriers for quercetin. Chitosan is a natural polysaccharide derived from the deacetylation of chitin. Positively charged CS‐NPs exhibit a strong affinity for the negatively charged cell membrane. This high‐affinity results in cell permeability and mucoadhesion [109].

Chemoresistance is a critical problem that occurs in various types of cancer. In a study on paclitaxel (PTX)‐resistant lung cancer cells, CS‐NPs were chosen for research. Quercetin‐chitosan nanoparticles (Qu‐CS‐NPs) demonstrated remarkable antitumor activities and effectively overcame the PTX resistance of lung cancer cells by inhibiting the AKT and ERK pathways. Moreover, cetuximab (Cet) has also been coated on Qu‐CS‐NPs and PTX‐CS‐NPs. Co‐treatment of Cet‐Qu‐CS‐NPs and Cet‐PTX‐CS‐NPs suppressed tumor growth in xenografts of PTX‐resistant lung cancer [14].

Moreover, in a tumor environment, the pH is lower than in normal tissue. pH‐sensitive nanoparticles can be utilized to regulate the release of anticancer drugs in the targeted environments. Combined Fe2O3, chitosan, and montmorillonite (MMT) offer several advantages. Fe2O3 exhibits magnetic properties that can be utilized for the targeted accumulation of the medicine, and the Fe2O3‐CS‐MMT nanocomposite controls the drug's release. The rate of quercetin release in a simulated tumor environment (pH = 5.4) was higher than in normal conditions (pH = 7.4) [110]. In addition, graphene oxide‐polyvinylpyrrolidone‐Fe2O3 (GO‐PVP‐Fe2O3), a magnetic nanocarrier, exhibited pH‐responsive behavior that controlled the release of quercetin in human breast cancer. It has been indicated that Qu‐GO‐PVP‐Fe2O3‐NPs have a high cytotoxic effect against human breast cancer cells [103]. Magnetic nanoparticles (MNPs) and pH‐sensitive nanoparticles can be administered in targeted cancer therapies. Combinatorial utilization of MNPs and pH‐sensitive nanoparticles can be highly efficient and advantageous.

### 8.2 Cancer Combination Therapies Using Quercetin

Many studies have shown that various combinations of quercetin with other anticancer drugs yield promising results and can benefit many cancers. Intra‐ and inter‐tumor heterogeneity is observed in tumors at the levels of genetics, histology, and cancer progression. These levels of heterogeneity result in tumor cell resistance and the evolution of their response to therapy. Therefore, combination therapies reduce drug resistance, improve treatment efficacy, and provide tumor cells with a broader range of cytotoxicity. Furthermore, multiple drugs used in combination therapies may exhibit synergy in cancer treatment [111]. Thus, quercetin seems a good option for combination therapies because of its anticancer and anti‐inflammatory properties [112]. Cetuximab, a monoclonal antibody that inhibits EGFR, is used in combination with quercetin to reduce tumor growth in paclitaxel‐resistant lung cancer cells and xenografts. Additionally, the combined administration of paclitaxel and quercetin suppresses cell growth and induces apoptosis. Also, quercetin inhibits the phosphorylation of AKT and ERK, thereby reversing paclitaxel resistance in lung cancer cells [14].

The combination of quercetin and 5‐fluorouracil (5‐FU) therapy inhibits the proliferation of hepatocellular carcinoma (HCC) cells [113]. Moreover, quercetin enhances the anticancer effects of 5‐FU in human colorectal adenocarcinomas. This combination increases the expression of PTEN, P53, and Bax and reduces the expression of Bcl‐2, which leads to suppressed cell proliferation and induced apoptosis [114].

In addition to all of these factors, we suggest incorporating quercetin into multiple drug therapies alongside other MerTK inhibitors. This approach will help address and mitigate the disadvantages and drawbacks of this group of inhibitors.

## 9 Limitations and Future Perspectives of Quercetin as a Complementary Option to Target MerTK in Cancer Therapies

Low solubility and instability in its free form are additional challenges that need to be addressed in future studies on the utilization of quercetin [103, 104, 105]. Another significant limitation is the potential drug interaction when administering quercetin and MerTK inhibitors. To address this concern, further experimental and computational studies should be conducted to investigate these interactions thoroughly.

It is necessary to conduct research to determine the optimal dosage of quercetin for use as an anticancer agent and the most effective dose range and frequency that can provide the maximum therapeutic benefits while minimizing potential adverse effects. Also, the expression of MerTK, like other genes, may vary between different types of cancer and even within specific tumors. Identifying patients who might benefit most from quercetin‐based therapy requires the development of reliable biomarkers or molecular profiling methods [115].

In to the bargain, clinical trials have not examined the safety, effectiveness, and long‐term implications of quercetin as a supplementary strategy for targeting MerTK in cancer therapy. It is crucial to understand the molecular processes underlying the interaction between quercetin and MerTK. Optimizing the use of quercetin in targeting MerTK will involve investigating the downstream signaling cascades and potential off‐target effects.

One of the main concerns in using quercetin in cancer therapeutic approaches is the scarcity of clinical trials [116]. Consequently, maintaining the balance between the efficient doses and toxic doses of quercetin due to its low absorption can be an important challenge in using this agent in anti‐cancer strategies. Despite quercetin's limited quantity of clinical trials, other flavonoids are used in several phase II and phase III clinical trials in combination with other therapeutics or as a single agent. Flavonoids demonstrate higher favorable results in lymphoid and hematopoietic tissues than in solid tumors. Inconclusive evaluations of the related clinical trials mostly stem from the intratumoral and intertumoral heterogeneity, patient collection data, and the wide range of drug administration forms [117]. Eventually, reducing the heterogeneity of patients and tumors and increasing the bioavailability of agents through drug delivery systems seem to be the essential approaches to enhance the outcomes of quercetin and other flavonoids' clinical trials.

Quercetin, a potential compound for targeting MerTK, may be a suitable candidate in cancer therapeutics alongside MerTK inhibitors (Table 3). However, further research is needed to fully understand its potential and ensure its effectiveness. Future clinical research should focus on pharmacokinetics, ideal dose, and long‐term safety.

**TABLE 3:** Efferocytosis consequences, MerTK inhibitors, and quercetin properties.

Efferocytosis	MerTK inhibitors	Quercetin
Anti‐inflammation	Excessive inflammation	Reduction of pro‐inflammatory factors
Recognition of apoptotic cells	Tumor cells evasion from apoptosis	Induction of pro‐apoptotic factors Inhibition of anti‐apoptotic factors
M2 polarization of macrophages	Autoimmunity	Improving CD8+ T, CD4+ T, and NK cells Suppression of regulatory T cells
Angiogenesis	—	Reduction in expression of VEGF‐A and HIF‐1α
Metastasis	—	Reduction of matrix degradation (MMP9 pathway) Downregulation of EMT mediators

## 10 Discussion

Natural substances are particularly effective against cancer. Numerous studies have examined how these compounds affect different signaling pathways, revealing their potential anticancer properties through various mechanisms, most notably epigenetic regulation [118, 119]. In this study, our intention was to gather data that demonstrates the influence of quercetin on MerTK and to clarify its effects on this particular pathway.

As an important point, the effects of quercetin on the suppression of CSCs' maintenance through several signaling pathways have been studied. There is a novel perspective that suggests this compound could be a suitable candidate for cancer combination therapies in conjunction with other anticancer agents. However, inhibiting the MerTK receptor as a potential anticancer therapy has presented numerous challenges. One of the main challenges in targeting TAM receptors is their structural homology, which can lead to toxicity and increased off‐target effects. Moreover, autoimmunity, resistance (due to “bypass signaling”), and excessive inflammation can occur when targeting more than one TAM receptor [42]. Another drawback that should be considered is the potential decrease in efferocytosis of tumor cells due to excessive inhibition of TAM receptors. This reduction in efferocytosis may contribute to the ability of tumor cells to evade apoptosis [120].

The existing challenges in utilizing MerTK inhibitors in cancer therapies motivated us to investigate the possibilities of using quercetin as a complementary factor (Table 3). Moreover, quercetin is a low‐cytotoxic compound that could be a favorable option in terms of cost‐effectiveness and enhancing the effectiveness of cancer combination therapies.

M2 tumor‐associated macrophages decrease antigen presentation, impairing the function of cytotoxic T cells (CTLs) [121]. As a result, tumor cells are able to evade recognition and lysis by the immune system. MerTK‐mediated efferocytosis promotes the M2 polarization of macrophages, possibly through the signaling of the STAT3 pathway [52]. M2 macrophages have higher expression levels of MerTK than M1 macrophages [121]. Moreover, it has been indicated that inhibiting MerTK significantly reduces the M2 polarization of macrophages [122]. Polarization of macrophages toward the M2 phenotype hinders the use of quercetin as an anticancer agent. Various studies have shown that treating cells with quercetin leads to M2 polarization in macrophages [123, 124]. Despite the therapeutic benefits of quercetin in various inflammatory diseases, the M2 polarization of macrophages induced by quercetin can be seen as a drawback in cancer treatments. Our suggestion is to promote M1 polarization of macrophages while utilizing quercetin in different cancer therapies.

On the other hand, combining quercetin with nanoparticles may not be the ultimate solution for complete suppression of MerTK in cancer therapies. Still, it could enhance the efficacy of this natural compound. Laboratory research and clinical trials are required to validate the prospects of combination therapies with quercetin and MerTK inhibitors on cancer patients.

In order to target cancer cells and get around the drawbacks of monotherapies, combination treatments employing quercetin and MerTK inhibitors provide a synergistic strategy. These results imply that quercetin can be a helpful adjunct therapy in clinical settings, particularly when combined with treatments that target MerTK.

There is hope that the development of new materials and methods to enhance the efficiency of quercetin will lead to advancements in reducing the harmful effects of MerTK in cancer. In addition, this deployment may alleviate the drawbacks of the MerTK inhibitors in the future.

This review presents a novel theoretical framework resulting from the synthesis of prior studies, positioning it not merely as a reiteration of existing information but as a strategic modulation point for future research. By integrating scattered results on quercetin's multifaceted interactions with MerTK and its downstream pathways, it offers a cohesive perspective that has not been clearly expressed in previous literature. This comprehensive style offers a forward‐looking trajectory for studies, paving the way for the plan of targeted combinational therapies in oncology.

## 11 Conclusion

Quercetin has anti‐inflammatory and anticancer effects, which makes it a potential adjuvant in combination therapy with MerTK inhibitors. The results of inhibiting numerous MerTK‐mediated signaling pathways, including JAK/STAT, PI3K/AKT, TGF‐β, NF‐κB, RAS, and SRC, are promising. Additionally, it reduces the upregulation of PD‐L1 and inhibits the interaction between PD‐1 and PD‐L1. This reduction can decrease metastasis and inhibit the survival and proliferation of cancer cells. The EMT program, the CSC phenotype, angiogenesis, and pro‐angiogenic factors can all be suppressed, while quercetin induces cancer cell death. These findings indicate that quercetin is a promising candidate for mitigating the adverse effects of MerTK inhibitors, such as heightened inflammation and evasion of tumor cell apoptosis. Therefore, conducting more studies, particularly clinical trials, and exploring solutions to overcome the limitations of its application, such as nanoparticle carriers, can enhance the effectiveness and reliability of this targeting approach. The utilization of quercetin for therapeutic approaches can be challenging; however, these issues may be resolved by combining it with MerTK inhibitors and integrating it into sophisticated delivery systems such as nanoparticles. Its effectiveness must be confirmed in future clinical trials in order to maximize its application in cancer treatments. The knowledge offered in this review provides the foundation for converting quercetin into a complementary therapy approach that is clinically meaningful.

## Author Contributions

S.J. searching, writing original draft, editing, and preparation of figures. S.G. conceptualization, investigation, supervision, writing review, editing, and preparation of figures.

## Ethics Statement

The authors have nothing to report.

## Conflicts of Interest

The authors declare no conflicts of interest.

## Data Availability

Data sharing is not applicable to this article as no new data were created or analyzed in this study.
